# Supplementary material for: OpenMM 7: Rapid development of high performance algorithms for molecular dynamics
Source: PLoS Comput Biol. 2017 Jul 26;13(7):e1005659. doi: 10.1371/journal.pcbi.1005659 (PMC5549999; doi:10.1371/journal.pcbi.1005659)
Supplement: S1 Source Code — (ZIP) [file pcbi.1005659.s002.zip › OpenMM-7.0.1-Source/docs/api-c++/generated/OpenMM.ThreeParticleAverageSite.html]

ThreeParticleAverageSite — OpenMM @OPENMM\_MAJOR\_VERSION@.@OPENMM\_MINOR\_VERSION@ documentation


# ThreeParticleAverageSite¶

*class* `OpenMM::``ThreeParticleAverageSite`¶
:   This is a `VirtualSite` that computes the particle location as a weighted average of three other particle’s locations. Assuming the weights add up to 1, this means the virtual site is in the plane of the three particles.

    Methods

    |  |  |
    | --- | --- |
    | `ThreeParticleAverageSite` | Create a new `ThreeParticleAverageSite` virtual site. |
    | `getWeight` | Get the weight factor used for a particle this virtual site depends on. |

    `ThreeParticleAverageSite`(int *particle1*, int *particle2*, int *particle3*, double *weight1*, double *weight2*, double *weight3*)¶
    :   Create a new `ThreeParticleAverageSite` virtual site. Normally the weights should add up to 1, although this is not strictly required.

        |  |  |
        | --- | --- |
        | Parameters: |  |

        - **particle1** – the index of the first particle
        - **particle2** – the index of the second particle
        - **particle3** – the index of the third particle
        - **weight1** – the weight factor (between 0 and 1) for the first particle
        - **weight2** – the weight factor (between 0 and 1) for the second particle
        - **weight2** – the weight factor (between 0 and 1) for the third particle

    double `getWeight`(int *particle*) *const*¶
    :   Get the weight factor used for a particle this virtual site depends on.

        |  |  |
        | --- | --- |
        | Parameters: |  |

        - **particle** – the particle to get (between 0 and `getNumParticles()`)

        |  |  |
        | --- | --- |
        | Returns: | the weight factor used for that particle |

High performance molecular simulation on GPUs


### Navigation

- Core Objects
- Forces
- Integrators
- Extras

©2015, Stanford University and the Authors.
|
Powered by Sphinx 1.3.1
& Alabaster 0.7.7
|
Page source
